# Geographic distribution of availability of adult day services in Missouri

**DOI:** 10.1093/geroni/igab046.2367

**Published:** 2021-12-17

**Authors:** Takashi Amano, Yung Chun, Sojung Park, Yi Wang

**Affiliations:** 1 Rutgers University-Newark, Newark, New Jersey, United States; 2 Washington University in St. Louis, St. Louis, Missouri, United States; 3 Washington university, St.Louis, Missouri, United States; 4 University of Iowa, Iowa City, Iowa, United States

## Abstract

Adult day service (ADS) is an important component of long-term supportive services. Geographic availability of ADS is an essential factor for aging in place especially for people with assistance needs. This study aims to examine the geographic distribution of availability of ADS and its relationship with the disadvantaged characteristics of neighborhoods. Data from the Missouri Department of Health and Senior Services and the American Community Survey were utilized. Geographic availability of ADS was measured as capacity (number of clients served) of ADS centers per week divided by the number of people who were 65 or older and under poverty at the census tract level. To examine neighborhood disadvantaged characteristics, principal component analysis was applied to construct a socioeconomic deprivation index (SDI). Using geographic information systems, we mapped ADS centers, geographic availability of ADS, and SDI scores. Pearson correlation coefficient was calculated between geographic availability of ADS and SDI scores. In 92.3% of the census tracts in Missouri, ADS centers are not available. Further, ADS centers are less likely to locate in rural areas or census tracts with higher numbers of residents 65 or older and poor. Also, lower availability of ADS was associated with higher levels of neighborhood disadvantage at a marginal level (r = - 0.163). Our findings suggested that strategies should be identified to provide ADS in rural areas, especially in the areas with higher levels of neighborhood disadvantage. Further investigation on the geographic distribution of ADS accessibility and its association with neighborhood characteristics is warranted.

